# A DIAGNOSTIC DILEMMA FOR A CHRONIC BLISTERING DISORDER

**DOI:** 10.51894/001c.123101

**Published:** 2024-08-30

**Authors:** Ishani Singh

**Affiliations:** 1 Internal Medicine Detroit Medical Center Sinai Grace

## 85

### INTRODUCTION

Bullous Systemic lupus erythematous (BSLE) is a rare condition that typically manifests as an acute vesiculobullous eruption in a patient known to have SLE. BSLE is more common in females. It usually occurs between the ages of 20-40.

### CASE DESCRIPTION

A 54-year-old female with a history of blistering skin disorder who presented after a fall. She reported extreme weakness and dizziness. She reported a history of blistering diseases. She was initially evaluated at a dermatology, and the biopsy showed deposition of C3 and IgG at the dermo-epidermal junction.On this admission, the patient was found to have superficial erosions in different stages on her trunk and extremities. She was found to have multiple intact tense bullae.In the past, she was treated with doxycycline and Bactrim and steroids which improved her symptoms. Her labs were pertinent high BUN/Cr which gradually worsened, ultimately requiring hemodialysis. Her renal ultrasound showed no hydronephrosis. Initially, it was thought that her kidney injury was due to dehydration; however, autoimmune causes were also considered as differential diagnoses. The initial ANA was negative and hepatitis panel were negative. Labs were pertinent for positive ANA the 2nd time with + fluorescein and titer of 1:1280, + Anti histone Ab, + ENA which were more consistent with lupus. She also then started on a steroid taper. Renal biopsy showed membranous glomerulopathy and ATN. Subepidermal blister biopsy results demonstrated bullous pemphigoid. The patient showed signs of clinical and laboratory improvement with steroids, dapsone 50 mg daily, and mycophenolate mofetil. She was discharged with a prednisone taper, triamcinolone, dapsone, and mycophenolate and was advised to follow up with rheumatology.

### DISCUSSION

SLE is a diagnosis of clinical and laboratory findings. Our patient was first diagnosed with bullous pemphigoid outpatient and later found to have symptoms and labs consistent with SLE due to her skin and kidney involvement. The case demonstrates the importance of taking a biopsy from a lesion or an intact vesicle, in any suspected patient with SLE. As the previous biopsy failed to give the exact results the possibility of not obtaining the tissue properly could have been a reason, as she was in her normal state.

